# Book Review: The Positivity Workbook for Teens: Skills to Help You Increase Optimism, Resilience, and a Growth Mindset

**DOI:** 10.3389/fpsyg.2021.713403

**Published:** 2021-07-05

**Authors:** Chris Fradkin

**Affiliations:** ^1^Psychological Sciences, University of California, Merced, Merced, CA, United States; ^2^Instituto de Psicologia, Universidade do Estado do Rio de Janeiro, Rio de Janeiro, Brazil

**Keywords:** positive psychology, PERMA, adolescents, Seligman, self-help

In *The Positivity Workbook for Teens: Skills to Help You Increase Optimism, Resilience, and a Growth Mindset*, the authors Goali Saedi Bocci and Ryan M. Niemiec deliver a self-improvement book for teens struggling with adolescence. The book is adapted from Martin Seligman's PERMA^TM^ model—a leading theory in positive psychology—which holds that flourishing is based on five pillars of well-being: positive emotions, engagement, relationships, meaning, and accomplishment (Seligman, 2011). The premise is to draw upon the strengths within the teen, to build self-confidence and bolster optimism.

The book starts with a description of the five pillars of well-being, followed by a *Signature Strengths Survey*. From a list of character strengths (Peterson and Seligman, 2004), the reader notes those strengths that best define them. These *signature strengths* provide fuel for the teen, to hone the skillsets they will need, to navigate the seas of adolescence.

Part 1, *Manifesting Positive Vibes*, draws on humor, hope, and zest, and helps the reader build positive emotions (“P” of PERMA^TM^). In Chapter 1, *Identifying Positive Emotions*, the reader reflects on the ten core positive emotions (joy, love, gratitude, interest, hope, amusement, pride, awe, inspirations, desire; Fredrickson et al., 2008, 2017), and gives examples of their presence in their life. In Chapter 2, *The ABCs of Mood*, the authors show the reader (a) how negative affect and cognitions can be modified through adaptive coping and behavior; and (b) how problem behaviors can be modified through adaptive affect and cognitions. In Chapter 3, *Forecasting Your Mood*, the reader considers several upcoming life events, within the framework of past successes and past failures.

In Chapter 4, *Happiness (Zest Booster!)*, the authors discuss hedonic (pleasure-based) and eudaimonic (meaning-based) routes to happiness. In Chapter 5, *Optimism (Hope Booster!)*, the reader is introduced to several different framing styles (internal, unstable, global vs. external, stable, specific) associated with optimism and well-being. In Chapter 6, *LOL and Live Fully (Humor Booster!)*, the authors tout the benefits of laughter in our lives (e.g., reduced blood pressure and stress hormones; Louie et al., 2016). In Chapter 7, *How Positive Emotions Can Relieve Mental Stress*, the authors offer coping skills to better manage stress.

Part 2, *Finding the Flow of Your Life*, draws on curiosity, creativity, and leadership, and helps the reader cultivate engagement (“E” of PERMA^TM^). In Chapter 8, *What Motivates You?*, the authors focus on intrinsic motivation—i.e., the undertaking of an activity for its inherent satisfaction (Ryan and Deci, 2000). In Chapter 9, *Getting in the Flow*, the authors talk about immersion in an activity. The reader revisits times when they were lost in such a flow, and notes the feelings and cognitions they inspire. In Chapter 10, *Engage with Your Signature Strengths*, the authors stress the importance of the reader's *signature strengths*.

In Chapter 11, *Commit Random Acts of Kindness*, the reader considers acts of kindness they can weave into their life. In Chapter 12, *Use Your Strengths to Engage Your Mind and Heart*, the authors differentiate between mind-oriented strengths (e.g., judgment, prudence) and strengths specific to the heart (e.g., love, kindness). In Chapter 13, *Overcoming Boredom*, the authors promote curiosity as an antidote for boredom. The authors have the reader focus their attention. This act is core to mindfulness, a practice with numerous health benefits (Fradkin, 2016, 2019d; Wu et al., 2019; Mazaheri et al., 2020).

Part 3, *Developing a Drama-Free (And Happy) Tribe*, draws on the strengths of love, kindness, and teamwork, and helps the reader nurture positive relationships (“R” of PERMA^TM^). In Chapter 14, *Next-Level Bully Management*, the authors warn the reader to think before they act, and make decisions from a place of inner peace. In Chapter 15, *Are You My Friend or Frenemy?*, the authors advise the reader to nurture their deep friendships, and be generous in expressing gratitude.

In Chapter 16, *Your Key Relationship Strengths*, the authors discuss honesty, love, forgiveness, kindness, humility, fairness, social intelligence, and zest. The reader reflects on what changes they can make to cultivate these strengths, with respect to their relationships with others. In Chapter 17, *Stop Comparing Yourself to Others!*, the authors suggest (a) setting social media limits, (b) engaging in noncompetitive activities, (c) focusing on the future, (d) practicing gratitude, and (e) remembering your character strengths. In Chapter 18, *Getting Off (or On) the Social Media Bandwagon*, the authors beg the reader to go “scroll-free” for a week, and record their observations in a journal. In Chapter 19, *Combatting Loneliness and Isolation*, the authors prescribe caring and knowing as a cure for loneliness.

Part 4, *Cultivating What Matters Most*, draws on gratitude, appreciation of beauty, and social intelligence, to help the reader find more meaning in their life (“M” of PERMA^TM^). In Chapter 20, *Savor Special Moments with Family*, the reader reflects on pleasant memories shared with family, and uses them as fuel to fuel the future. In Chapter 21, *Build an Attitude of Gratitude*, the authors have the reader write a letter to someone important in their life, and thank them for their support and influence. This practice—expressive writing (Pennebaker, 1997)—is core to many therapeutic programs (see Fradkin, 2019a,b,c), and shows benefits in a multitude of areas (Pennebaker et al., 1988, 1989, 1990). In Chapter 22, *Create Mindfulness Moments of Strength*, the authors introduce the practice of mindfulness (Bluth, 2020). Studies find that mindfulness practice reduces stress, anxiety, and depression (Fradkin, 2017a, 2020; Borquist-Conlon et al., 2019), and bolsters resilience and self-worth (Fradkin, 2017b; Mazaheri et al., 2020).

In Chapter 23, *Mindful Walking in Nature*, the authors ask the reader to take a walk through the forest, and while doing so keep their senses open. Awareness of this type is important with this practice, and with the many other variants of mindfulness awareness (Bluth, 2020; Fradkin, 2021). In Chapter 24, *Awe and Elevation*, the reader (a) writes about three beautiful things they came across that day, (b) creates a portfolio of things that trigger inspiration, and (c) adds beauty to their personal environment (e.g., plants, photographs). In Chapter 25, *Cultivating Spirituality and the Sacred*, the reader considers (a) the purpose of their life, (b) the meaning of spirituality, and (c) what connects them to the sacred in their life. Chapter 26, *Overcoming Jealousy, Envy, and FOMO*, is tailored for the teen that overloads themselves—trying to keep up with their peers—and is driven by the fear of missing out (FOMO).

Part 5, *Your Goals, Your Life*, draws on perseverance, perspective, and zest, to help the teen along the path to their accomplishments (“A” of PERMA^TM^). In Chapter 27, *Your Best Possible Self*, the teen envisions themselves one year in the future, having reached their full potential or a milestone. They ask themselves, What character strengths are needed to make this a reality? In Chapter 28, *Hope for Your Goals*, the teen picks a goal, and using *pathways thinking*, conceives several different routes to take them there. In Chapter 29, *Taming Your Inner Critic*, the authors introduce the teen to loving-kindness meditation, a practice potent in promoting positive emotions (Fredrickson et al., 2017; Fradkin, 2019e).

Part 6, *Healthy Body, Healthy Mind*, taps self-regulation, prudence, and perseverance, to link a healthy body to a healthy mind. In Chapter 30, *Promoting Good Health*, the authors introduce the Five Pillars of Good Health (Niemiec, 2019): exercise, healthy sleep, healthy eating, social activity, and self-regulation to good health. The reader identifies which pillars are strong within themselves, and which pillars may need reinforcement. The reader then brainstorms a plan to reinforce the pillars that need work.

*The Positivity Workbook for Teens* is a self-improvement book for teens struggling in the throes of adolescence. It is grounded in the teachings of positive psychology (Seligman, 2011). There are exercises gleaned from evidence-based practices. The approach is age-appropriate. The writing is precise. The PERMA^TM^ structure offers a sound foothold. In sum, *The Positivity Workbook for Teens* is an engaging self-help program for teens, their families, and their counselors.

## Author Contributions

The author confirms being the sole contributor of this work and has approved it for publication.

## Conflict of Interest

The author declares that the research was conducted in the absence of any commercial or financial relationships that could be construed as a potential conflict of interest.
